# Kinetically Controlled Synthesis of Highly Emissive Au_18_SG_14_ Clusters and Their Phase Transfer: Tips and Tricks

**DOI:** 10.1021/acsomega.2c07663

**Published:** 2023-02-08

**Authors:** Chengjie Wang, Hairong Zhao, Zhongsheng Ge, Lizhuang Dong, Xiao Han, Avula Balakrishna, Praveen Kumar Balguri, Yixi Wang, Udayabhaskararao Thumu

**Affiliations:** † Institute of Fundamental and Frontier Sciences University of Electronic Science and Technology of China, Chengdu 610054, China; ‡ Department of Aeronautical Engineering, Institute of Aeronautical Engineering, Hyderabad 500043, India

## Abstract

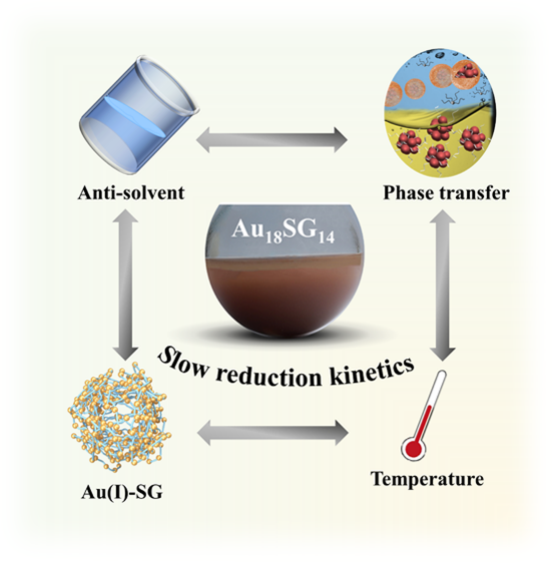

Glutathione (GSH) protected gold nanoclusters (Au_*n*_SG_*m*_ NCs) have been attractive because of their novel properties such as enhanced luminescence and band gap tunability at their quantum confinement region (below ∼2 nm). Initial synthetic routes of mixed-size clusters and size-based separation techniques had latter evolved toward atomically precise nanoclusters via thermodynamic and kinetic control routes. One such exemplary synthesis taking the advantages of a kinetically controlled approach is producing highly red-emissive Au_18_SG_14_ NCs (where SG = thiolate of glutathione), thanks to the slow reduction kinetics provided by the mild reducing agent NaBH_3_CN. Despite the developments in the direct synthesis of Au_18_SG_14_, several meticulous reaction conditions still need to be understood for the highly adaptable synthesis of atomically pure NCs irrespective of the laboratory conditions. Herein, we have systematically studied a series of reaction steps involved in this kinetically controlled approach starting from the role of the antisolvent, formation of precursors to Au-SG thiolates, growth of Au-SG thiolates as a function of aging time, and exploring an optimal reaction temperature to optimize the desired nucleation under slow reduction kinetics. The crucial parameters derived in our studies guide the successful and large-scale production of Au_18_SG_14_ at any laboratory condition. Next, we investigated the effect of pH on the NCs to study the stability and the best suitable condition for the phase transfer of Au_18_SG_14_ clusters. The commonly implemented method of phase transfer at the basic conditions (pH > 9) is not successful in this case. However, we developed a feasible method for the phase transfer by diluting the aqueous NC solution to enhance the negative charges on the NCs’ surface by increasing the degree of dissociation at the carboxylic acid group. It is interesting to note that after the phase transfer, the Au_18_SG_14_-TOA NCs in toluene as well as in other organic solvents exhibited enhanced luminescence quantum yields from 9 to 3 times and increased average photoluminescence lifetimes by 1.5–2.5 times, respectively.

## Introduction

Atomically precise thiolate-protected ultrasmall gold nanoclusters (Au_*n*_SR_*m*_ NCs, SR = alkyl thiolate; *n* and *m* are the numbers of respective species) of smaller sizes (*n* < 20) stabilize by unique atomic packing. Often, these NCs are the metastable species, taking part to build the bigger NCs in a desired synthesis (*n* < 20); for example, Au_15_(SR)_13_ and Au_18_(SR)_14_ NCs impart an active role in the growth of bigger Au NCs (e.g., Au_25_SR_18_). In general, their structure is composed of a smaller inner core (below 10 Au atoms) protected by unique types of gold-thiolate motifs that provide quite strong interaction among the inner core and the outer staple motifs. As a result of this strong interaction, there is a smaller atomic distance in the Au_motif_–Au_core_ bond for Au_18_SR_14_ (3.09 Å) compared to Au_25_SR_18_ (3.16 Å). Because of these increased interactions among the core and the ligands, in these ultrasmall NCs, especially in the water-soluble NCs, the optical properties are highly affected by the type of ligand (e.g., a red shift of 50 nm from −SG to cyclohexane thiolate protected Au_18_ NCs).^1^ In these water-soluble systems, the core is protected by electron-rich thiolates that often alter the optical properties for good reason such as enhanced photoluminescence (PL) with a quantum yield of 3–8%. For example, the clusters Au_22_SG_18_, Au_18_SG_14_, and Au_15_SG_13_ (SG = thiolate of glutathione) are highly emissive yet possess inherent biocompatibility and have created enormous interest from fundamentals to applications in drug delivery, sensors,^2^ catalysis,^3^ energy harvesting,^4^ and bioimaging.^5^ The most widely used applications are size-specific and have greater potentiality when their sizes are small. Moreover, biology and photocatalyst applications are related to their PL emission; interestingly, the water-soluble Au NCs surpass the emissive properties of organic-soluble NCs.^6^ The reason for the enhanced fluorescence in the case of water-soluble NCs over the others is the charge transfer from the ligand through the Au–S bond and the direct donation of the delocalization of the electrons from the electron-rich groups of the ligands to the Au core.^7^

There have been several tens of well-defined Au NCs existing in the literature; among them, a handful of clusters have gained more special attention than others by considering at least two key factors, namely, thermal stability^8,9^ and superior luminescence properties.^10−13^ For example, irrespective of the type of thiolate, the Au_25_ NCs proved to carry higher thermal stability and hence gave an opportunity for a greater extent of studies, but this cluster still suffers from a poor PL quantum yield (PLQY 0.1%).^14,15^ Thus, the core size and structure are also important to tune the PLQY to higher levels. For example, for the same capping agent of glutathione, the NCs of Au_15_, Au_18_, Au_22_, and Au_23_ exhibit superior fluorescence with QYs of 4.0, 6.8, 2.5, and 1.3%, respectively. These values are much higher than those of the Au_25_ and Au_38_ NCs (QY <0.1%).^16^ Thus, the best combination of size of a cluster core and the glutathione’s electron-donating capability aids to enhance the luminescence in these NCs.

Because of the immense interest, various synthetic methodologies have been developed for the precise control of ultrasmall water-soluble Au_*n*_SR_*m*_ NCs.^17^ Indeed, SG-protected NCs are the initial examples to explore these novel optical properties of gold at the quantum confinement region.^18,19^ Initial efforts of synthetic methods by Whetten *et al.* and Tsukuda *et al.* yielded mixed-size clusters of Au_*n*_SG_*m*_ NCs, which were subjected to polyacrylamide gel electrophoresis (PAGE) for size-selective separation processes.^20,47^ Later, many research groups predominantly focused on the one-pot synthesis of these NCs at atomic precision, which reduces several tedious size separation steps. In fact, the one-pot synthetic chemistry developed is specific to one specific NC, and a completely different size-controlled method for other NCs is needed. Specifically, there are robust synthetic routes available for larger-sized NCs (number of atoms ≤25). Typically, these synthetic methods to obtain NCs with molecular purity utilize the principles of thermodynamic control of a reaction. They mainly include two-step size focusing methods (polydisperse NCs to monodisperse NCs)^21^ such as thiol-mediated thermal etching reactions.^9^ In the first step, the polydisperse NCs are produced by the reduction of Au(I)thiolate by a strong reducing agent (e.g., sodium borohydride (NaBH_4_) and maintenance of the reaction parameters at size-focused conditions (temperature, excess thiols, etc.). During this process, the bigger NCs turn to thermodynamically stable NCs, whereas the smaller NCs sacrifice their identity. Thus, the two-step size-focusing approach is probably not safe to apply for small NCs because of their heavily exposed inner core toward the surroundings. Moreover, these are considered as the nearest nanostructures to the Au(I)-thiolate complexes; hence, they decompose to the thermodynamically stable Au(I)-thiolate complexes.

As a result, it has been a huge challenge for chemists to develop methods for these ultrasmall NCs. Researchers have developed different types of “kinetically trapped approaches” for these thermodynamically less stable NCs. They include low-temperature synthesis, reactions conducted at slow reduction kinetics,^22^ and solid-state routes.^23,24^ Among them, the solution-mediated one-pot synthesis dictated by slow reaction kinetics is promising toward less stable NCs for facile and high-yield synthesis. specifically, an exemplary synthesis of well-defined glutathione-protected Au_18_ NCs taking advantage of a slowdown in gold ion reduction kinetics with a controlled nucleation-based growth process using the mild reducing agent NaBH_3_CN. Note that NaBH_3_CN is a mild reducing agent compared to commonly used NaBH_4_ for the reductive decomposition of Au(I) thiolate complexes.^1,13^ As noticed in the earlier section, Au_18_ is one of the smallest clusters and stays at the top in terms of their better emissive properties in the red region compared to others.^13^ However, the majority of water-soluble NCs studied for their size transformations,^25,26^ surface sensitive issues concerning the pH conditions,^10,27,28^ phase transfer to organic solvent for enhancing the luminescence are limited to bigger systems (*n* < 20).

Overall, only a handful of reports on Au_18_(SG)_14_ are available on these NCs by various pioneer research groups such as those of Tsukuda *et al.*,^20^ Pradeep *et al.*,^13^ Stamplecoskie and Kamat,^29^ and Xie *et al.*(30) and the reports on NCs with other ligands by Jin *et al.*,^31^ Zhu *et al.*,^1^ and Sau *et al*.^32^ Additionally, there are limited studies on the theoretical understanding of these NCs by Tlahuice and Garzon^33^ and Tang and Jiang^34^ to predict the [Au_18_(SR)_14_] structure to be a prolate bitetrahedral Au_8_ core capped by two dimers (-SR-Au-SR-Au-SR-) and two trimers (-SR-Au-SR-Au-SR-Au-SR-). The first Au_18_(SG)_14_ NCs were isolated by Negishi *et al*.^20^ through gel electrophoresis from their crude synthesis that contains mixed-size clusters. Various research groups had followed this route for various studies such as mass spectral analysis and homogeneous catalysis of chemoselective hydrogenation of nitrobenzene derivatives^35^ and explored its application as a photocatalyst. Stamplecoskie and Kamat^29^ studied the photocatalytic reduction activities of several clusters (Au_10_SG_10_, Au_15_SG_13_, Au_18_SG_14_, and Au_25_SG_18_); interestingly, the Au_18_SG_14_ showed stronger light-absorbing properties compared the others together with a higher efficiency of electron transfer; hence, this cluster is considered as the best sensitizer for light-harvesting applications compared with the rest of NCs (Au_10–12_, Au_15_, Au_25_).

First, successful one-pot synthesis of these Au_18_(SG)_14_ NCs was obtained in a solution phase through slow reduction kinetics by Pradeep *et al.*,^13^ who studied their extensive mass spectral analysis on this system. Yao *et al.*(36) recently developed a similar direct kinetically controlled approach where pH regulation is used as a tool to target the composition specifically of Au_18_(SG)_14_ NCs. Later, Zhu *et al.*(1) succeeded in obtaining their single crystals after the postsynthetic thiolate-mediated phase transfer of aqueous Au_18_SG_14_ NCs to an organic medium. A breakthrough in the structural determination of these NCs reveals interesting atomic packing in these NCs that carry a bi-octahedral Au_9_ inner core staple by three Au(SR)_2_, one dimeric Au_2_(SR)_3_, and one tetrameric Au_4_(SR)_5_ motif.

These structural details along with property studies had raised special attention to an in-depth understanding of their intriguing optical properties. Especially, the enhanced emissive properties of Au_18_SG_14_ promise potential applications in catalytic and light-harvesting and biological applications. So, the ability to attain size-pure and high-yield synthesis is important for the widespread use of this material. In contrast, there are only a few studies on emphasizing the crucial synthetic parameters of this highly sensitive and less stable Au_18_(SG)_14_. For example, in the literature, for the Au_18_SG_14_ NCs, slightly different absorption profiles are seen (Figure S1) irrespective of the similarity in the synthesis method.

Although there are many mechanistic studies related to size-focusing routes (solvent nature, role of Au-SG complexes, reaction time, reaction temperature, etc.),^37^ such studies are rare in the case of slow reduction nucleation and growth processes. In fact, from our practical experience, as well as in agreement with this community of researchers, the NC synthesis is highly sensitive to parameters such as room temperature, humidity conditions (typically based on the weather), and reaction time at each deciding stage that affect the synthesis of a desired NC. With this intention, herein, we extended our studies to highlight the crucial parameters involved in this slow reduction kinetic approach of Au_18_SG_14_ by taking an example of a reaction conducted in an aqueous medium where the reductive decomposition of Au-SG is performed by NaBH_3_CN. For several optoelectronic applications (light-emitting device, luminescent sensors), requires the NCs soluble in organic solvents with greater PL quantum yields.^38^ During phase transfer from water-soluble to organic-soluble, it is important to keep the core structure integrity. However, the phase transfer of Au_18_SG_14_ clusters is very sensitive, and the clusters change their core structure during the phase transfer. Thus, we have developed suitable phase transfer methods for these water-soluble Au_18_SG_14_ clusters to an organic medium (toluene) by taking advantage of previous transferring methods.^39^ The optical properties of these organic-soluble Au_18_ clusters are further studied.

## Results and Discussion

**Figure 1:**
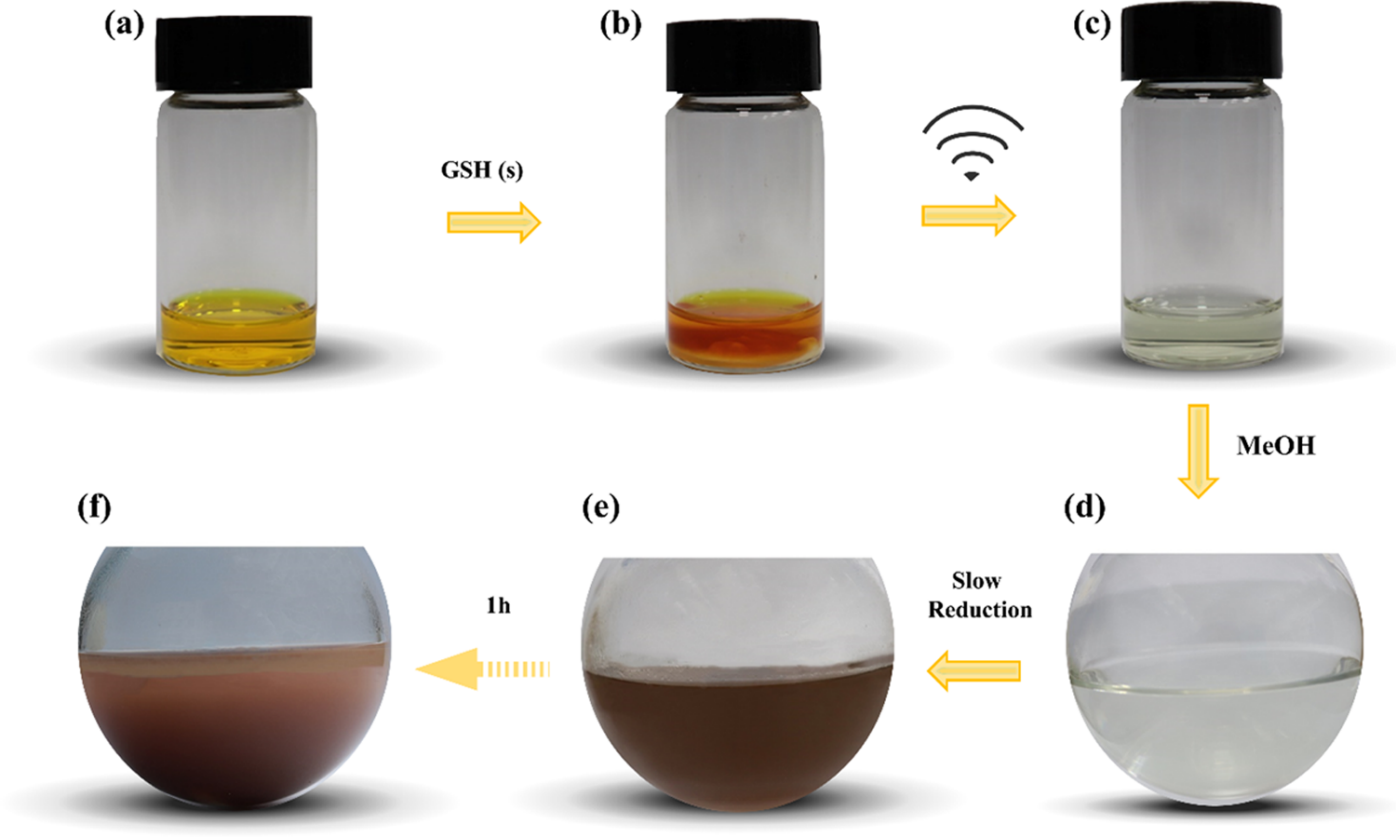
Demonstration of a one-pot synthesis route for Au_18_SG_14_ NCs conducted at 37 °C. (a) HAuCl_4_·3H_2_O in methanol/water (1:2) solution. (b) Addition of solid GSH to solution (a). (c) Sonication of solution (b). (d) Addition of excess methanol to solution (c). Aqueous NaBH_3_CN is added to solution (d) for initiating the reduction process. Photographs of the (e) immediate and (f) after an hour reactions are presented.

The synthesis of Au_18_SG_14_ NCs was performed under ambient conditions without inert gas protection. Synthetic details and procedure of these NCs are given in the Experimental Section (SI) and Figure 1. In brief, a concentrated gold chloride solution is prepared in a methanol/water (1:2) mixture (Figure 1a); to this solution, a required amount of solid GSH is added. Immediately, the solution color change changes from yellow to dark orange yellow (Figure 1b). These color changes indicate that only a partial amount of GSH reacted with the gold salt; hence, the Au(I)-SG complexation has not occurred immediately, which can be identified clearly by the disappearance of its color. Typically, a colorless Au-thiolate complex formation occurs in the presence of excess thiols (above 1:3 ratio of Au/SG).^40^ Next, the solution is subjected to sonication for 2 min, resulting in the degradation of the dark orange yellow solution to a nearly colorless solution. The colorless solution indicates the formation of Au(I)-SG complexes (Figure 1c). Next, the solution is diluted with 45 mL of methanol where the reaction solution is dominated by an excess amount of methanol (water/methanol, 1:48). This methanolic Au(I)-SG solution is kept under stirring for 10 min (Figure 1d). It is crucial to limit the stirring time below 10 min for the quality synthesis of NCs. After 10 min of Au(I)-SG formation, an aqueous NaBH_3_CN is introduced in few steps. The color changes from colorless to light brown within a few minutes of the introduction of the reducing agent (Figure 1e). Over time, the clear solution turns toward red color turbidity as a result of the coagulation process of freshly born nanoclusters due to the presence of antisolvent methanol. After stirring for an hour, NCs come out of solutions as red precipitates as a result of methanol used as a part of the reaction solution (Figure 1f). The presence of an antisolvent in the reaction process contributes to the controlled growth kinetics for the precise synthesis of Au_18_SG_14_ NCs. The crucial role of methanol in the successful synthesis of Au_18_ NCs was confirmed by a control experiment where the synthesis was performed using fully deionized water. The resulting NC solution does not yield the Au_18_SG_14_ NCs but clusters with different compositions as confirmed by the deviation from the characteristic absorption profile and shows no red emission from these NC solutions (Figures S2 and S3).

**Figure 2:**
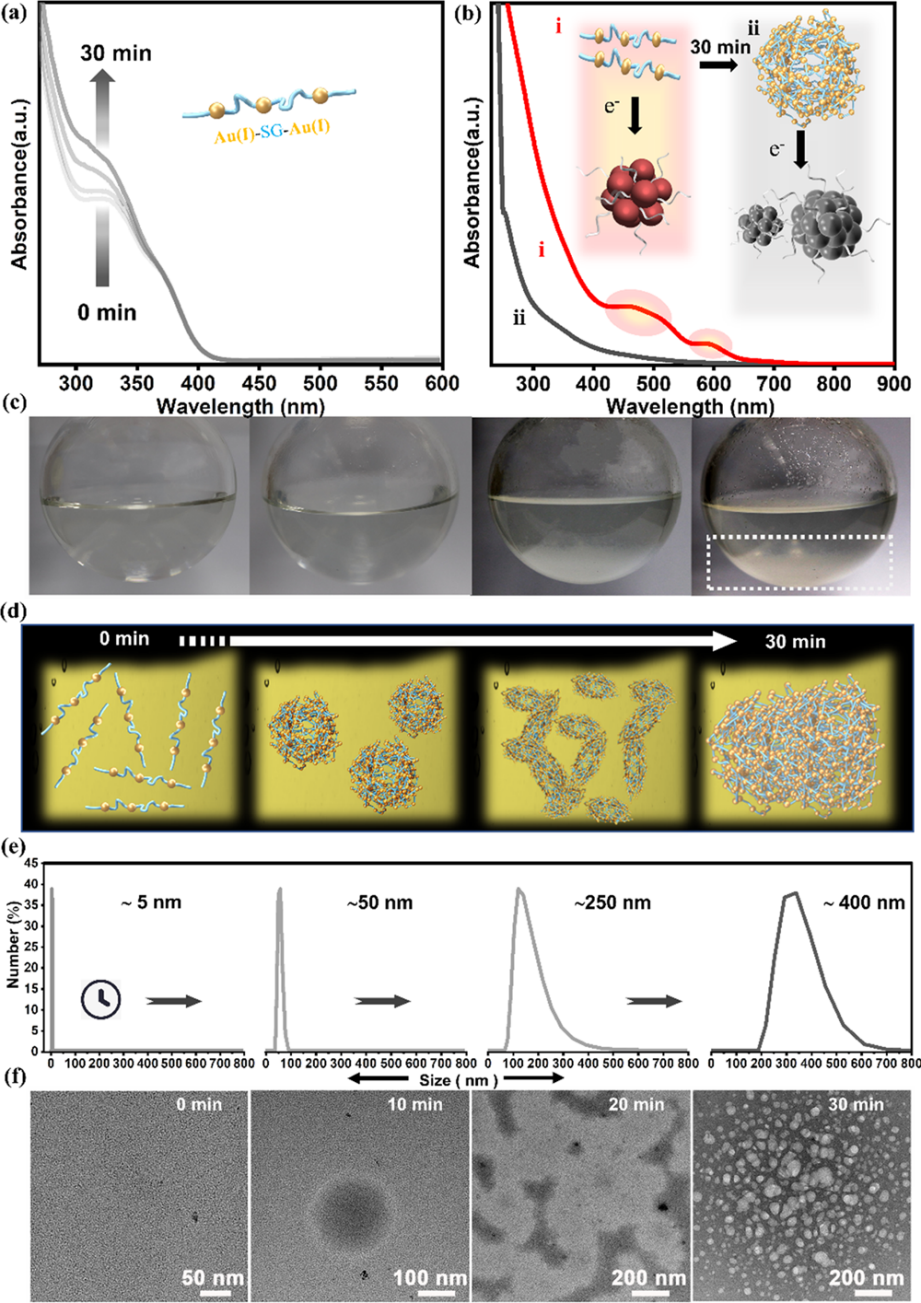
Effect of aging on Au(I)-SG complexes in the methanolic solution. (a) The time-dependent absorption spectra of the Au(I)-SG solution during the aging time from 0 to 30 min. The inset shows the schematic representation of a single unit of the Au-SG complex. (b) The absorption spectra of the Au_18_SG_14_ NCs synthesized at different Au(I)-SG aging times. The reduction was conducted when Au(I)-SG was aged for (i) 10 min and (ii) 30 min. The inset shows the schematic representation of the aging effect on the final sizes of NCs. (c) Photographs of the reaction solution collected at different aging time intervals. The gray-colored dotted box represents the white precipitate of [Au(I)-SG]_*x*_ aggregates. (d) Schematic representation of the Au(I)-SG growth process was deduced on the basis of the time-dependent (e) DLS and (f) TEM analyses.

However, we found that one needs to be watchful at some of the crucial steps involved in the synthesis of Au_18_SG_14_. Otherwise, it could cause miscellaneous results in terms of the product yield with diversified emissive properties from batch to batch. Thus, the following discussion includes the essential precautions needed to achieve high-yield Au_18_ NCs with the best emissive properties from the synthesis. Another important factor is the stirring time of Au(I)-thiolate complexes in methanolic solution. The absorption spectra of the Au(I)-SG complexes during their aging time are presented in Figure 2a. The peak at 330 and 375 nm is related to the Au(I)-SG complex,^41^ which is gradually changed over time, indicating that the overall monomeric Au(I)-SG complex undergoes self-aggregation toward [Au(I)-SG]_*x*_. There is an increase in turbidity in the reaction mixture as a result of this aggregation, which also causes a hyperchromic shift in the UV–vis absorption spectrum with time.^41^ Furthermore, we have compared the absorption spectra of the NCs synthesized at two different time intervals of aging thiolate; *i.e.*, aqueous NaBH_3_CN is added to the solution containing two different degrees of [Au(I)SG]_*x*_ aggregation. Figure 2b shows the absorption spectra of the NCs synthesized at 10 and 30 min of aging [Au(I)SG]_*x*_ under agitation by magnetic stirring. The appearance (Figure 2b,i) of broadband absorption at 515 and 590 nm indicates that the 10 min of [Au(I)SG]_*x*_ aging time is ideal for the synthesis of Au_18_ NCs, whereas the extended stir times result in a featureless absorption profile that indicates the formation of mixed-size NCs (Figure 2b, ii). Because it is an influential factor for the Au_18_ NC synthesis, we further analyzed the solution with respect to the stirring time. The photographs of the solutions at different aging times of [Au(I)SG]_*x*_ complexes are compared. A transparent solution is seen at the initial stages of Au(I)SG complexes and is transformed to white turbidity over time (Figure 2c). Time-dependent dynamic light scattering (DLS) studies reveal that the aggregate size increased from ∼5 to ∼400 nm gradually (Figures 2d,e). This clearly indicates that the degree of polymerization of [Au(I)SG]_*x*_ complexes increased over time, and those structures were further studied by transmission electron microscope (TEM) analysis. TEM was carried out on the solution containing [Au(I)SG]_*x*_ complexes at different time intervals. The TEM analysis (Figure 2f) of the initial stages (0 min) of the Au(I)-SG solution shows the presence of tiny particles of sizes ∼2–3 nm. These tiny nanoparticle-like morphologies could be due to e-beam reduction of Au(I)SG molecules. At extended aging times, the Au(I)SG molecules transfer to micron-sized [Au(I)SG]_*x*_ spherical structures that then aggregated to larger amorphous structures. At 30 min of aging time, the [Au(I)SG]_*x*_ networks become thick, and the electron beam could not pass through this material (Figure 2f). At this stage, a white [Au(I)SG]_*x*_ sediments out from the solution (Figure 2c, indicated by the dotted box). The relation between the size of [Au(I)SG]_*x*_ aggregation and the quality of NCs after the slow reduction process is obvious. Previously, Jin^42^ reported that the stirring speed influences the size of [Au(I)SG]_*x*_ aggregates and the overall NC synthesis process of Au_25_ NCs. Other than a few such reports, in the majority of the reactions with fast reduction kinetics (in the case of a strong reducing agent, NaBH_4_), the aging step is not very crucial for the size control of the NCs. Hence, we emphasize that the size of [Au(I)SG]_*x*_ is a crucial factor for the slow reduction kinetics approach. In this case, the size of the [Au(I)SG]_*x*_ aggregate had a substantial influence on the strength of reduction performed by the mild reducing agent (NaBH_3_CN) that could eventually alter the nucleation and growth process, which decides the final composition of the NCs. The take home message is that one must be cautious; it is important to limit the size of the [Au(I)SG]_*x*_ aggregate via maintaining a proper aging time (below 10 min) to obtain the high-yield synthesis of Au_18_SG_14_. Otherwise, the same synthesis route could lead to mixed-size clusters at an extended aging time of [Au(I)SG]_*x*_ (above 10 min).

Next, we have noticed another hurdle for the reproducible synthesis of high-yield Au_18_SG_14_ NCs, *i.e.*, reaction temperature, which poses challenges to the synthesis. For the same synthesis path, the NC growth kinetics is affected by changes in seasons (summer (37 °C) and winter (17 °C) in Chengdu, China) and subsequent changes in laboratory temperatures. In fact, the synthesis reproducibility is highly influenced by the seasons of the year (especially summer versus winter). From our practical observation, the color of the NC crude solution synthesized in winter was different from that of the synthesis conducted in the summer. The nucleation and growth kinetics of Au_18_SG_14_ synthesis is slower than the traditional reduction process (reducing agent NaBH_4_) that is heavily influenced by the temperature of the reaction. This led us to study several controlled experiments related to the temperature, which reveal that the season had a significant influence on the synthesis path and, consequently, the yield and emissive properties of Au_18_SG_14_ NCs.

**Figure 3:**
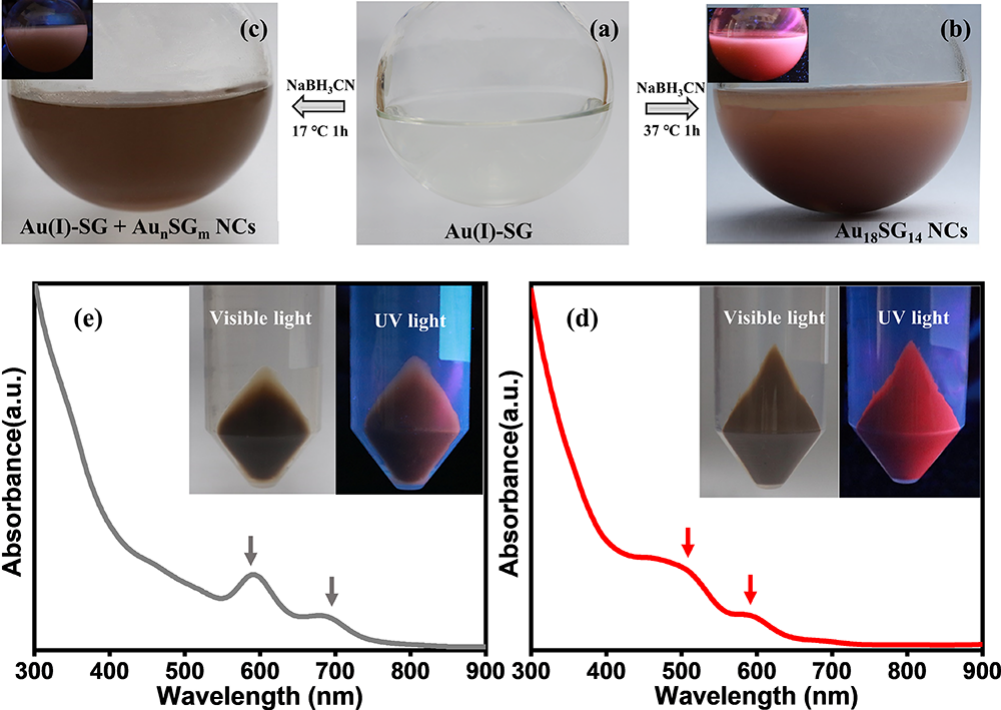
The influence of reaction temperature on the kinetically controlled synthesis of Au_18_SG_14_ NCs. (a) The Au(I)-SG complex in methanolic solution. Addition of aqueous NaBH_3_CN at two different reaction temperatures: (b) 37 °C and (c) 17 °C. The insets of (b) and (c) are the respective solutions photographed under UV light. (d, e) Absorption spectra of the NCs synthesized at 37 and 17 °C, respectively. The insets show the respective solid pellet under visible light and UV light.

Figure 3 shows the collective differences of pristine NCs prepared while the reduction process was maintained at two different temperatures (37 and 17 °C). This is done by placing the Au(I)-SG complex solution at the preplanned temperatures immediately after its formation (Figure 3a). Figure 3b,c shows the digital photographs collected in the daylight. There are obvious color differences such as reddish brown and brownish black for the crude NC solutions made at 37 and 17 °C, respectively. There is a striking contrast in their emission behaviors as seen in their photographs taken under UV light (insets of Figure 3b,c). The crude solution of the NCs prepared at a relatively higher temperature (37 °C) exhibits a strong red emission, whereas the other NC solution shows a poor emissive nature. The NCs can be separated easily by centrifugation as they are soluble in water, whereas the thiolates are insoluble. After centrifugation, the precipitate appeared as a pellet at the bottom part of the centrifuge tube. Strikingly, for the low-temperature synthesis, the yield of this cluster product is poor, but a majority still stay as unreacted [Au(I)-SG]*x* thiolates as an impurity. This implies that partial reduction takes place at these temperatures. The insets of Figure 3e and Figure S4, at the edge of the crude NC pellet, show the presence of white [Au(I)SG]_*x*_ precipitate that is absent in the case of crude NCs prepared at higher temperatures. For comparison, precipitates (insets of Figure 3d,e) of these samples are presented under visible and UV light. The emissive behaviors of the NC products prepared at 37 °C show strong red emission in both colloidal and solid states, whereas weak emissions are observed for the other case. Note that the emission originated from the Au_18_SG_14_ NCs that are dominant in the former case. The UV spectra of the purified NCs prepared at both reaction conditions are presented in Figures 3d,e; those contrast with each other. The absorption profiles of the NCs synthesized at a higher temperature (37 °C) show prominent absorption bands at 590 nm (2.1 eV) and a broad band at 515 nm (2.4 eV) (Figure 3d). The yield of Au_18_SG_14_ clusters in terms of Au is 64%, starting from 150 mg of HAuCl_4_·3H_2_O, which is slightly more than the previous reports on Au_18_SG_14_.^1,13^Figure 3e show the UV spectrum of the NCs formed at 17 °C, which shows the presence of sharp absorption peaks at 590 nm (2.1 eV) and 690 nm (1.8 eV). This absorption profile is different from that of the original Au_18_SG_14_ NCs discussed before. The absorption at 590 nm is present but with a different peak width compared to that of the original Au_18_ NCs. In addition, the notable differences are an additional peak at 690 nm and the absence of a characteristic broad band at 515 nm. These indicate that the NCs produced at the low-temperature synthesis did not correspond to Au_18_SG_14_ NCs but were of a different Au_*n*_SG_*m*_ NC composition (composition yet to be identified). The reduction process initiated by NaBH_3_CN occurred at low temperatures, facilitating the diversified kinetic trapping of metastable NCs more than the reaction at higher temperatures. These results demonstrate that the ideal temperature for high-yield precise control of Au_18_SG_14_ synthesis needs to be around 37 °C.

After the successful synthesis of Au_18_SG_14_ NCs by considering these three important factors, i.e., the role of antisolvent, Au-SG aging time, and reaction temperature (37 °C), we emphasize the phase transfer of these clusters from an aqueous to organic medium. The phase transfer method was previously implemented in the case of Au_25_SG_18_^28^ and Au_22_SG_18_^10^ using the phase transferring agent TOAB.^43^ Sau *et al*.^32^ also transferred the Au_18_ clusters protected with 3-mercaptopropionic acid (MPA) ligand using TOAB. This intact state of the NCs can be understood by comparing the UV spectra before and after phase transfer, which need to be the same in both cases. Typically, TOAB is highly soluble in toluene and could be dissociated into a TOA^+^ cation and Br^–^ anion at the toluene–water interface. The cation TOA^+^ electrostatically interacts with the anionic carboxylate group of the glutathione ligand on the NC surface from the water medium, which results in an induced hydrophobic nature around the NC and it not being soluble in water anymore. Thus, the NC undergoes phase transfers from an aqueous to organic medium. This technique is simple and straightforward to transfer the NCs whose ligands carry a carboxylate group at the end of the alkyl chain as shown by a previous report by Sau *et al*. on Au_18_(SR-COOH)_14_ NCs.^32^ However, the method requires further optimization depending on the type of passivating ligand on NCs. Because glutathione exists in a deprotonated form containing both −COO^–^ and −NH_3_^**+**^ moieties (zwitterion ion form), it could alter the electrostatic attraction force among TOA^+^ and anionic NCs.

**Figure 4:**
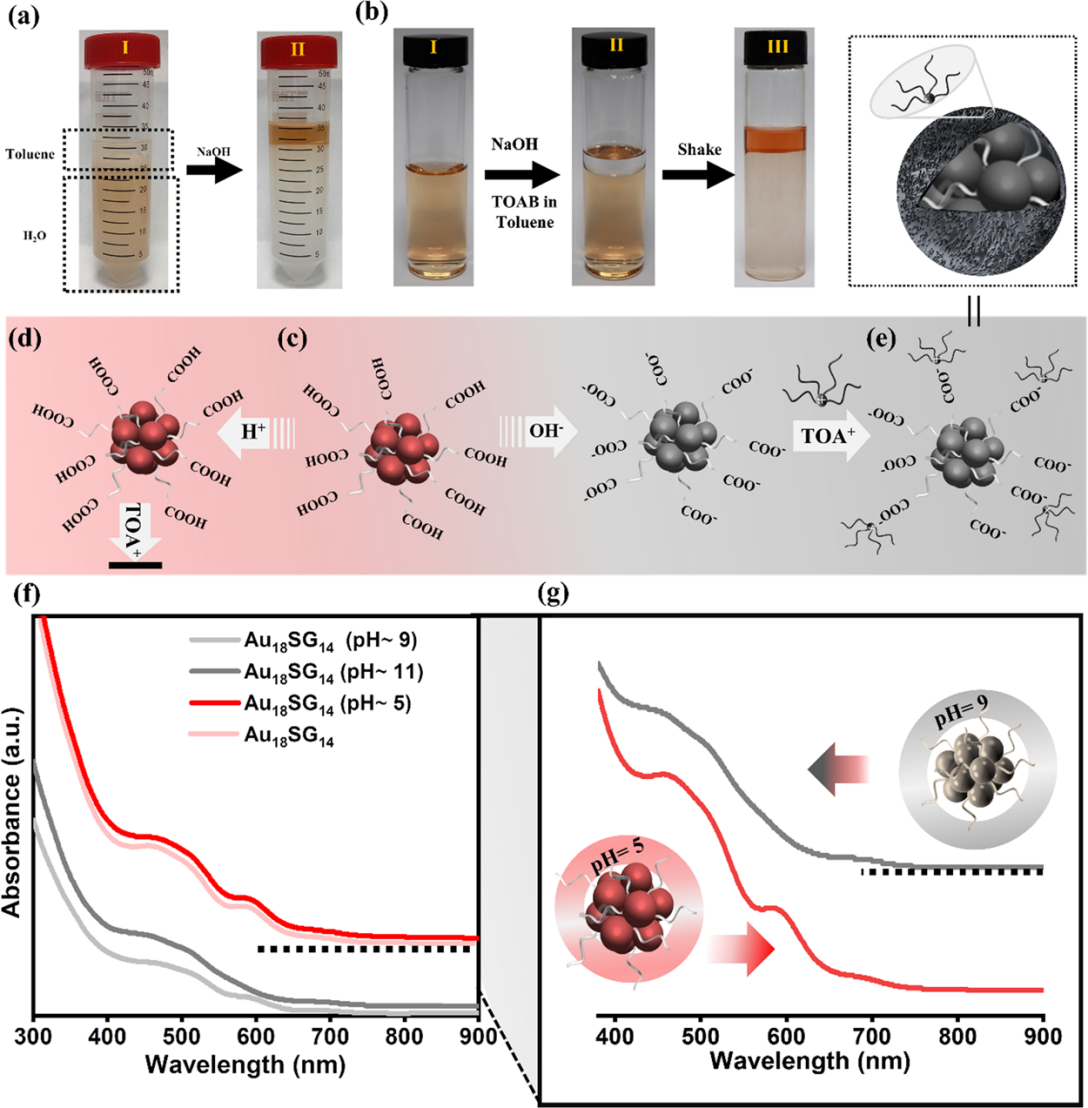
The influence of pH on the phase transfer of Au_18_SG_14_ NCs from aqueous to toluene. (a) The unsuccessful phase transfer of Au_18_SG_14_ NCs under nearly neutral conditions (I), undergoes phase transfer after the addition of base (II). (b) Aqueous Au_18_SG_14_ NCs (I) at basic conditions (II) are subject to successful phase transfer to toluene, which contains TOAB (III). Schematic representation of Au_18_SG_14_ NCs (c) in an aqueous medium subjected to (d) basic and (e) acidic conditions. The color changes of NCs from red to gray indicate the structural decomposition of Au_18_SG_14_ in basic conditions. The arrow in (d) indicates the unsuccessful phase transfer of NCs. (f, g) The absorption spectra of the Au_18_SG_14_ NCs at different pH conditions.

The next section deals with the critical factors needed for the successful phase transfer of Au_18_SG_14_ NCs. Fundamentally, the active center that leads this process is the charge state of COOH, which can be either a neutral or negative charge upon deprotonation. The negative charge state (COO^–^) is crucial for the phase transfer of the NCs in this process. When this phase transfer reaction was performed on aqueous Au_18_SG_14_ (without additional NaOH), it could leave the −SG ligands with a nearly neutral charge and was not feasible for electrostatic attraction. The NCs themselves are unsuccessful in phase transfer in the absence of NaOH, and toluene is colorless even though the phase transfer reaction was allowed to continue overnight (Figure 4a, I). For this reason, typically, NaOH is introduced to the reaction, which stimulates the whole process by deprotonation of the −COOH group, leading to the formation of −COO^–^ (Figure 4d), which enhances the electrostatic interaction between the TOA^+^ cation resulting in the facile transfer within 1–2 min, and the toluene solution turns to red (Figure 4a, II). This methodology of performing the reaction at basic conditions is successfully implemented in the case of Au_22_(SG)_18_ and Au_25_(SG)_18_.^10,28^Figure 4b shows the process photographs of the NCs subjected to phase transfer based on previous methods (typically, performed at basic conditions). Figures S5 and S6a present the absorption spectra of the NCs before and after phase transfer performed based on previous methods (performed at basic conditions).^10^ The data clearly indicate that the characteristic absorption band at 590 nm in the aqueous medium disappears after the phase transfer to toluene. We attributed these changes to the result of structural or charge state modifications in an NC. This implies that in contrast to the highly stable Au_25_SG_18_ and Au_22_SG_18_ systems,^10^ the phase transfer of Au_18_SG_14_ is quite challenging and requires further optimization.

Certainly, the main emphasis of retaining the core intact without charge or structural changes during the phase transfer at basic conditions did not serve the purpose. Nevertheless, the core changes during the reaction were not related to the influence of the TOAB but the interaction with OH^–^. Figure 4f,g shows the influence of pH on the stability of Au_18_SG_14_ studied by UV–vis spectroscopy. For these experiments, the acidic and basic pH is adjusted by acetic acid and NaOH. At acidic conditions (pH = 5), the UV spectra remain the same as those of the original Au_18_SG_14_ solution. In acidic conditions, the thiolate exists in a protonated form containing −COOH, causing a strong hydrogen bonding among the NCs, which does not facilitate the phase transfer (Figure 4d). In contrast, at moderate basic conditions (pH = 9), the spectra started to decline in the absorption peak located at 590 nm. At increased basic conditions (pH = 11), a complete disappearance of the absorption band at 590 nm is clearly noticeable. The peak at 515 nm is not affected by the basic conditions but only the peak at 590 nm. We infer that the strong nucleophilic nature of the OH^–^ species that increases the surface negative charge (Figure 4e) eventually undergoes slight modification to the atomic packing leading to these absorption changes. These structure-altered NCs were phase transferred to toluene. Figure S6b shows the UV spectra of structure-altered NCs in different solvents such as toluene, DCM, acetone, THF, and methanol, and they show similar absorption profiles.

**Figure 5:**
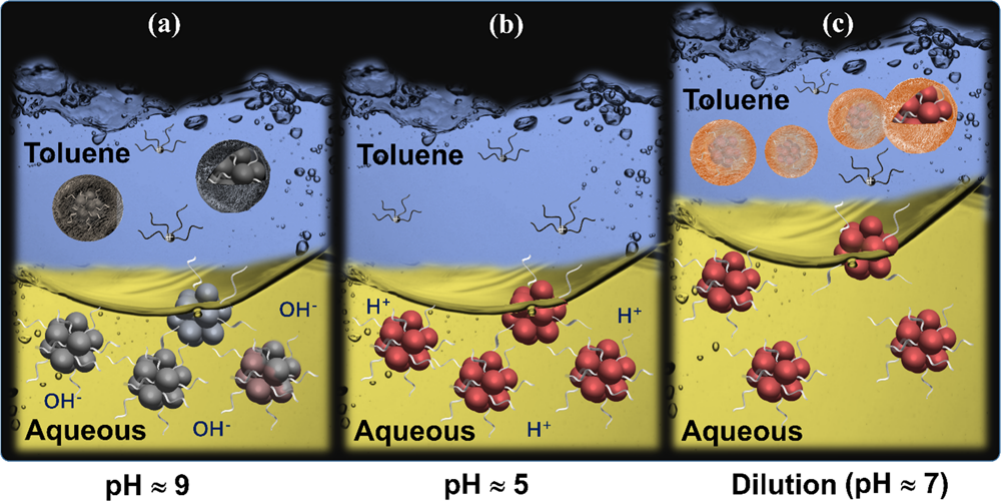
Summary of the phase transfer process at (a) basic, (b) acidic, and (c) dilution methods.

Overall, the phase transfer process is summarized in Figure 5. Figure 5a shows that phase transfer occurs but there is a change of Au_18_SG_14_ clusters’ core at alkaline conditions, whereas at acid conditions, the Au_18_SG_14_ clusters are stable but phase transfer is not observable (Figure 5b). To eliminate the subversive effect of OH^–^ caused by basic conditions, one needs to search for alternative routes. The usage of excessive TOA^+^ in the toluene phase helps the phase transfer without altering the absorption peak position, but the resulting NCs are unstable because of excessive ions in solutions. In addition, it is hard to separate the excess TOA^+^ ions from the NCs because of the higher solubility of TOA^+^ in most of the common organic solvents.

**Figure 6:**
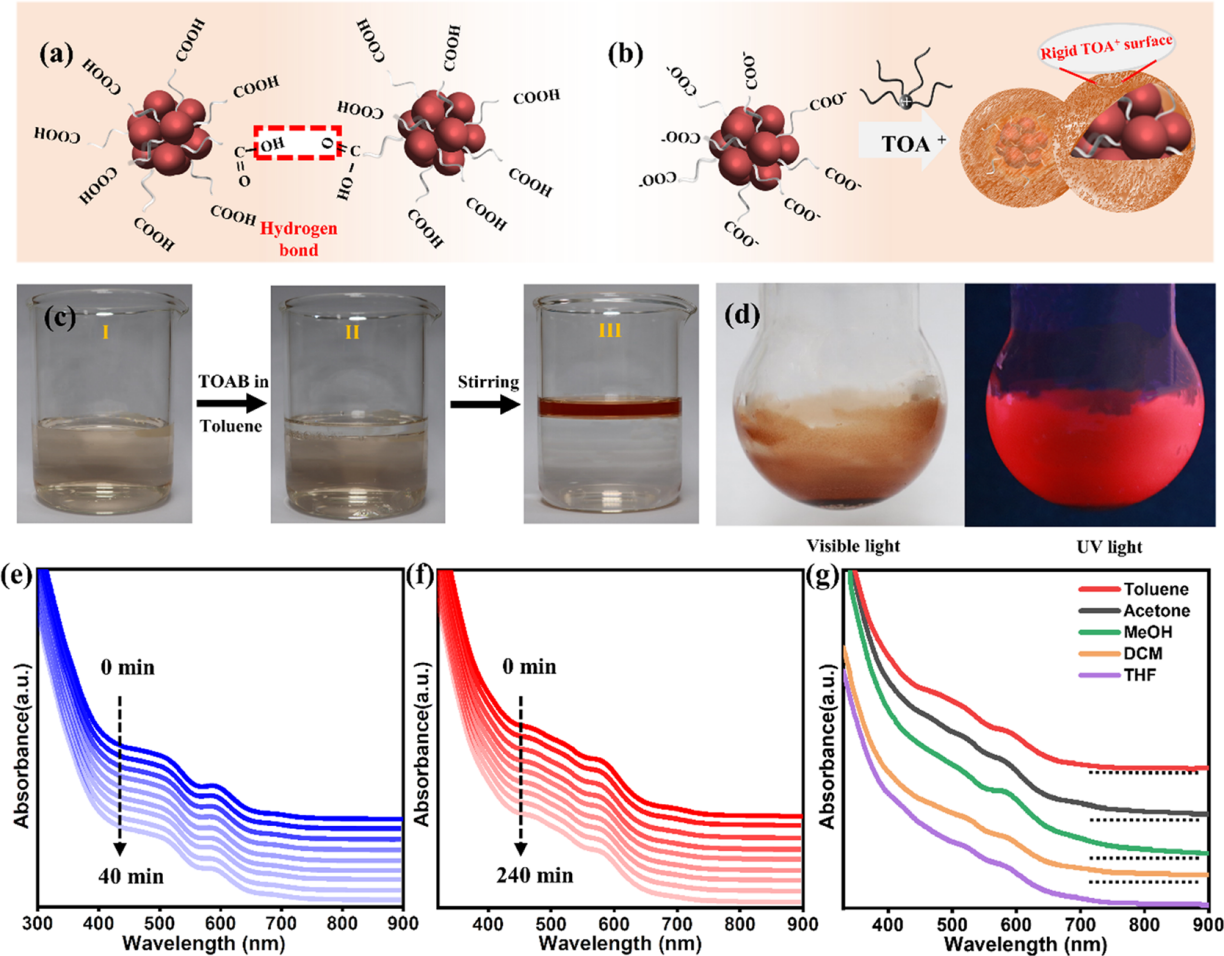
(a–c) The adaption of the dilution method for the successful phase transfer of Au_18_SG_14_ NC from aqueous to toluene. (d) The photographs of the Au_18_SG_14_-TOA NC powders under visible and UV light. The time-dependent absorption spectra of (e) Au_18_SG_14_ NCs in water and (f) Au_18_SG_14_-TOA NCs in toluene. (g) The absorption spectra of the Au_18_SG_14_-TOA NCs in different organic solvents.

To keep the Au_18_SG_14_ NCs intact after phase transfer, we searched for another method. In this route, the aqueous NC solution was diluted with more water to supply additional energy to carry out the dissociation of the −COOH group (Figure 5c). It is well known from the Ostwald dilution law that the degree of dissociation increases with dilution.^44^ In addition, dilution separates the NCs apart, which can reduce the intermolecular hydrogen bonding among individual Au_18_SG_14_ NCs. These interactions are strong in case of the zwitterion form of ligands on the NC surface. Figure 6a schematically illustrates that −COOH will form a hydrogen bond between the Au_18_SG_14_ clusters, which brings clusters together and hinders the phase transfer of NCs. In this method, the Au_18_SG_14_ solutions were diluted 8 to 10 times with water to enhance the number of anionic carboxylate groups on Au_18_SG_14_ (Figure 6b and c, I). Figure S7 shows that the zeta potentials (ζ’s) of Au_18_SG_14_ in concentrated and dilute solutions are −2 ± 3 and −25 ± 5 mV, respectively. It also proves that more anionic carboxylate groups are present on Au_18_SG_14_ upon dilution. Note that the NC solution in concentrated form does not exhibit a strong negative charge (nearly zero, −2 ± 3 mV), which could be due to the zwitterion form (both positive and negative charges) of the glutathione on the NC surface. To this, 20 mL of toluene with TOAB was added (Figure 6c, II). After stirring for several minutes, the toluene layer changed from colorless to red, which shows that the phase transfer occurred successfully (Figure 6c, III). The absorption does not undergo any changes, indicating that the NCs are intact without charge and structural modifications (Figure S8). This blue shift (∼10 nm) is attributed to the presence of TOA^+^ bound to the terminal carboxylate anions of the cluster ligands in the toluene phase. The solid samples of Au_18_SG_14_-TOA can be acquired upon the removal of toluene through a rotary evaporator, and they exhibit stronger red emission (Figure 6d) compared to the Au_18_SG_14_ powders.

Figure 6e shows the time-dependent absorption spectra in water at room temperature. The peak at 590 nm has declined to 90% compared to the initial spectrum over 40 min. Figure 6f shows the time-dependent UV spectra of Au_18_SG_14_-TOA NCs in the toluene obtained from the dilution method. The same 90% decline of the peak at 590 nm took 6 times more time (240 min) than the aqueous soluble Au_18_SG_14_. It also can infer that the stability of Au_18_SG_14_-TOA in toluene is better than that in water. Figure 6g shows the absorption spectra of Au_18_SG_14_-TOA in different solvents such as toluene, DCM, acetone, THF, and MeOH, which show similar absorption profiles. Interestingly, the absorption spectra of Au_18_SG_14_-TOA in methanol show a ∼5 nm red shift compared to that of other organic solvents, which is similar to that of Au_18_SG_14_ in water. This indicates that the polarity-based solvent pattern causes changes in the optical properties.

**Figure 7:**
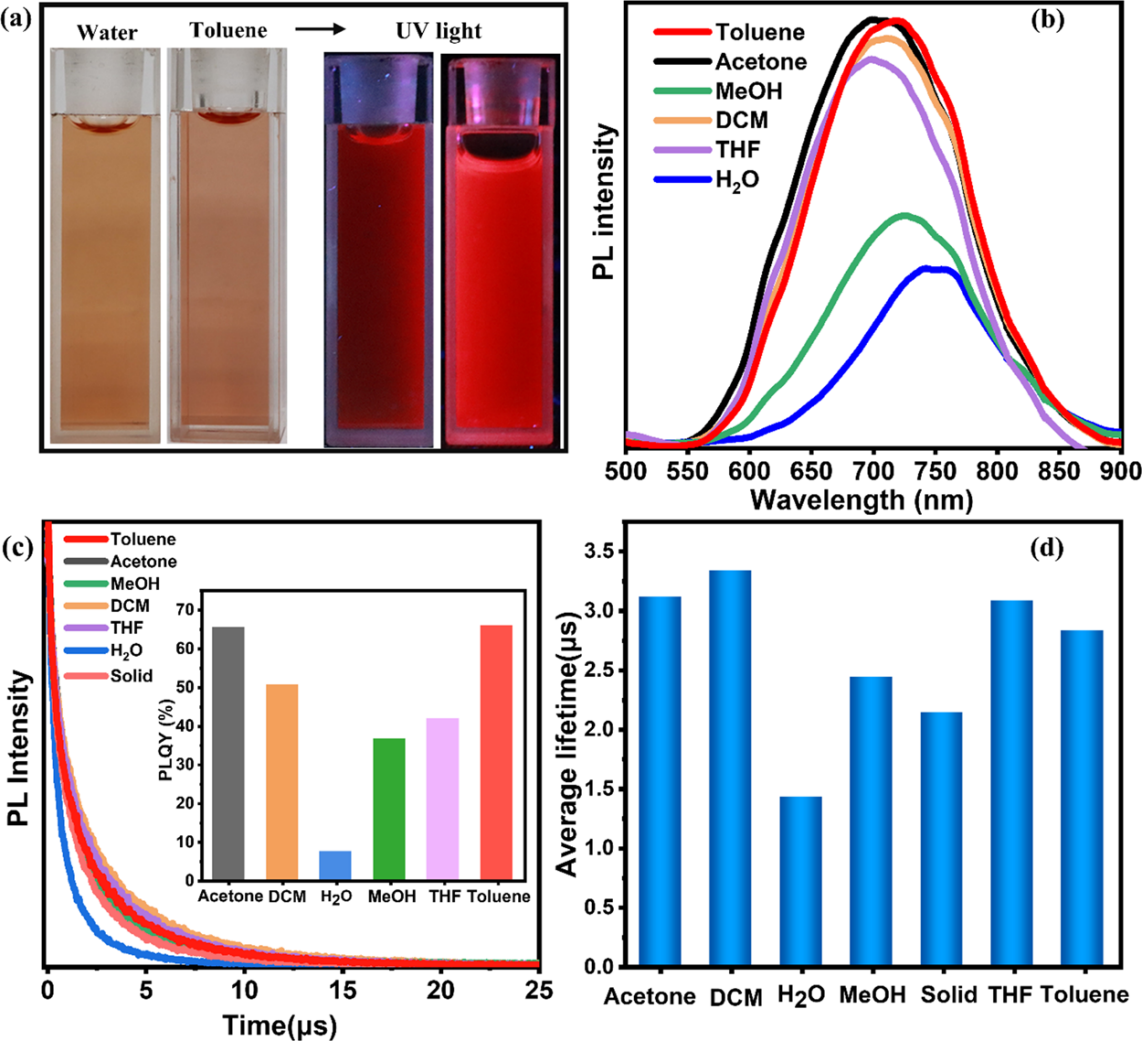
(a) Photographs of the Au_18_SG_14_ and Au_18_SG_14_-TOA NCs are compared under UV and visible light. (b) PL spectra and (c) luminescence decay profiles of NCs are compared in different solvents. The inset of (c) shows the quantum yield of Au_18_SG_14_ NCs in different solvents. (d) The average lifetime values of NCs in different solvents are compared. Note that the NCs in water are measured before phase transfer and the rest are measured after this process.

Interestingly, after the successful phase transfer of Au_18_SG_14_ to the toluene phase, the Au_18_SG_14_-TOA NCs’ red emission properties are enhanced substantially. This increased red emission intensity is visible to the naked eye as shown in the photos collected under UV light (Figure 7a). Similar luminescent enhancement is observed in the case of Au_22_SG_18_ clusters^45^ and others.^11^ Lee *et al.*(10) attributed this excellent luminescence enhancement after the phase transfer to the rigidity of the gold shell covered around the Au_22_SG_18_ clusters (Figure 6b). We further studied the influence of solvents on the luminescence properties of Au_18_SG_14_-TOA clusters (Figure 7b). The PL spectrum of Au_18_SG_14_ in H_2_O shows a red emission wavelength at 745 nm, which matches well with the previous reports.^1,12,13^ At the same concentration of Au_18_SG_14_, the PL emission of NCs in toluene got enhanced along with a blue shift in the peak maxima to 720 nm (Figure 7b).

Next, the PL properties of the Au_18_SG_14_-TOA clusters of the same concentration are studied in different solvents (H_2_O, methanol, toluene, THF, DCM, acetone). The results clearly show that organic solvents had a key role in increasing the fluorescence intensity and emission wavelength. The PL intensity of NCs is in the following order: toluene, acetone, DCM, THF, methanol, and water. The luminescence peak position is blue-shifted for other solvents when compared to the aqueous solutions. Interestingly, all other organic solvents (both polar and nonpolar) show a similar trend of a blue shift in the emission peak located at 700–720 nm. But the Au_18_SG_14_-TOA NCs in methanol behave similarly to Au_18_SG_14_ NCs in water, although there is a slight blue shift in PL spectra in the case of NCs in methanol. Note that there are similarities in their absorption profiles too as discussed earlier. The PL quantum yield of the Au_18_SG_14_-TOA NCs in methanol is nearly 3 times more than that of the aqueous soluble NC and 3 times less than that of NCs in toluene. The similar optical properties of NCs in water and methanol could be due to the higher freedom of TOA^+^ in MeOH (relative polarity of 0.76). This could allow the release of TOA^+^ from the Au_18_SG_14_-TOA NCs and might behave in a dynamic equilibrium between bonding state and free state.

We also compare the Au_18_SG_14_ luminescence decay profiles of the NC solutions before and after phase transfer. Figure 7c shows that the luminescence decay was fitted with a biexponential function, and a conclusion can be made that the luminescence decays more rapidly in H_2_O than other solvents. This indicates the lower nonradiative decay nature in the case of organic solvents. The rhodamine 6G (QY = 0.59) in water is used as the standard to measure the quantum yield (QY).^46^ The inset in Figure 7c shows the QY of Au_18_SG_14_ clusters in different solvents. It shows that after phase transfer to toluene, the quantum yield improved from 7.78 to 65.9%. The intensity increased nearly 9-fold. The QY increasing trend is in the following manner: toluene > acetone > DCM > THF > methanol > H_2_O. The shift in the photoluminescence (PL) maxima and increase in the PL intensity are attributed to the reduction in the nonradiative recombination process (as seen from the lifetime data) due to the change in the cluster and solvent interactions as a result of the rigidity of the shell formed by the phase transfer agent covered around the NCs. The changes in the PL position from one solvent to another depends on the relative separations among ground and excited states and the solvent stabilization strengths of ground and excited states.^11^

Figure 7d shows the average lifetime of Au_18_SG_14_ clusters in different solvents measured at room temperature; from an aqueous to organic medium, the lifetime values are increased by more than 2-fold (from 1.25 to 2.7 μs). The large lifetime in the microsecond range along with the large Stokes shift suggests that the emission from Au_18_SG_14_-TOA NCs is related to phosphorescence whose efficiency became doubled after the phase transfer.

**Figure 8:**
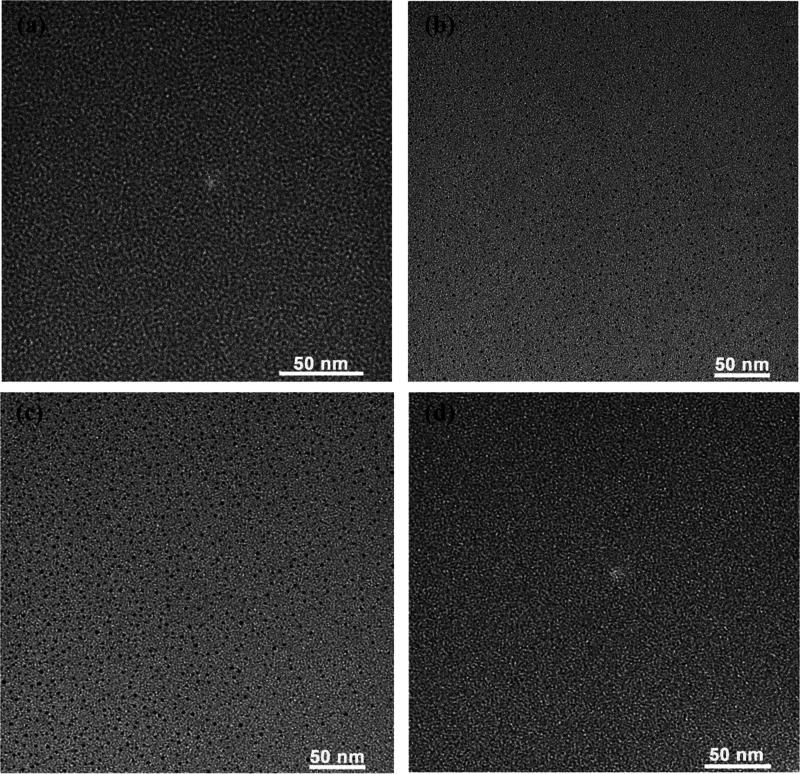
TEM of Au_18_ NCs in different solvents. (a) Au_18_SG_14_ clusters in H_2_O. (b) Au_18_SG_14_-TOA NCs in THF. (c) Au_18_SG_14_-TOA NCs in acetone. (d) Au_18_SG_14_-TOA NCs in toluene.

In general, it is hard to observe the NCs in TEM measurements because of their tiny crystallinity. Here, we studied the dispersion of Au_18_SG_14_ and Au_18_SG_14_-TOA NCs within a solvent by TEM to find the best suitable solvent to view these NCs. For these measurements, nearly the same concentration is maintained. The solid Au_18_SG_14_-TOA is dissolved in different solvents. Figure 8 shows the TEM images of the NCs measured in different solvents, and all of them contain the presence of NCs of size around 1–2 nm. We have not noticed any kind of aggregation in the NCs. Among all, the NCs in acetone and THF are clearly visible. In other cases, it is hard to observe because of the low TEM contrast with the background for these NCs. Hence, we suggest that THF and acetone are the best solvents to view the solvent without much difficulty.

## Conclusions

In summary, we have provided crucial factors that influence the growth kinetics for the highly adaptable synthesis of Au_18_SG_14_ NCs. Especially, these NCs are possible to synthesize only through a slow nucleation and growth process by maintaining the presence of an appropriate amount of antisolvent and slow-reducing agent. Thus, unlike the traditional fast reduction process kinetics, this process was particularly impacted by the size of the Au(I)-SG complex and the reaction temperature during the reductive decomposition process of these thiolates. We also showed the influence of pH on the stability of the NCs, where acidic pH is favorable for its stability; in contrast, the cluster composition undergoes changes at basic conditions. The drawback of the subversive effect of these OH^–^ ions on Au_18_SG_14_ hinders the typical phase transfer methods. Furthermore, we have shown that the solvent dilution method is optimal; hence, the Au_18_SG_14_ clusters can transfer to the toluene phase easily. Furthermore, because of the induced rigidity around the NCs’ outer layer by TOA^+^, the phase-transferred Au_18_SG_14_-TOA NCs show a blue shift in luminescence with predominant luminescence enhancement and increased average lifetimes compared to the original water-soluble Au_18_SG_14_ NCs. We also found a polarity-based solvent pattern effect on the optical properties of Au_18_ NCs. We believe that this work opens new opportunities as a result of the roadmap in the synthesis and optimal phase transfer methods for these highly luminescent Au_18_SG_14_ NCs to be used in optoelectronic applications.

## Supporting Information Available

Synthesis procedures related to the NCs and the phase transfer methods; UV–vis spectra of the NCs at various controlled experiments (PDF)

## Dedication

Dedicated to Professor Thalappil Pradeep on the occasion of his 60th Birthday.
