# Supplementary material for: Kinetically Controlled Synthesis of Highly Emissive Au18SG14 Clusters and Their Phase Transfer: Tips and Tricks
Source: ACS Omega. 2023 Feb 8;8(7):6884–94. doi: 10.1021/acsomega.2c07663 (PMC9948219; doi:10.1021/acsomega.2c07663)
Supplement: Supplementary file 1 — ao2c07663_si_001.pdf [file ao2c07663_si_001.pdf]

Supporting Information

Kinetically Controlled Synthesis of Highly Emissive $Au_{18}SG_{14}$ Clusters and its Phase Transfer: Tips and Tricks

Chengjie Wang[1], Hairong Zhao[1], Zhongsheng Ge[1], Lizhuang Dong[1], Xiao Han[1], Avula Balakrishna[1], Praveen Kumar Balguri[2], Yixi Wang[1], Udayabhaskararao Thumu[1,*]*

[1] Institute of Fundamental and Frontier Sciences University of Electronic Science and Technology of China, Chengdu 610054, China

[2] Department of Aeronautical Engineering, Institute of Aeronautical Engineering, Hyderabad 500043, India

Experimental Section

Materials: All the following chemicals were commercially available and were used without further purification. $HAuCl_4.3H_2O$, $NaBH_3CN$ and AR grade solvents, methanol (MeOH), dichloromethane (DCM), tetrahydrofuran (THF), acetone, and toluene are purchased from Adamas. Rhodamine 6G is purchased from HEOWNS. Tetraoctylammonium bromide (TOAB, 98%) is purchased from Bide-Pharma-tech. Reduced GSH (γ-Glu-Cys-Gly, molecular

weight of 307) were purchased from Sigma-Aldrich. Deionized water (18.2 MΩ cm) was used in our experiments.

Synthesis of $Au_{18}SG_{14}$: The original synthesis was reported by Atanu *et al.* and modified the reaction temperature as per our required study. In typically synthesis of $Au_{18}SG_{14}$ clusters, about 150 mg of $HAuCl_4.3H_2O$ (solid) was added to 1 mL MeOH, following the addition of 2 mL distilled water to form $HAuCl_4$ (1MeOH: $2H_2O$) solutions. 300 mg GSH was added to above prepared $HAuCl_4$ solutions. This solution was subjected to ultrasonication for several minutes to transform the color of the solution from yellow to colorless. Next, the solution was diluted by addition of 45 mL of MeOH. The stirring time is crucial for the high yield synthesis of $Au_{18}SG_{14}$, which is around 5 to 10 mins (further discussions are given in the main text). In order to study the effect of reaction temperature, two such solutions were placed at two different reaction temperatures such as 17 °C and 37 °C, respectively. After that 4.5 mL of aqueous $NaBH_3CN$ solution was added stepwise by 1 mL pipette (5-6 times) and continued the stirring for 1 hour. Next, these formed nanoclusters were subjected to purification process. The $Au_{18}SG_{14}$ NCs were precipitated out from the solution through centrifugation process. The precipitate is further washed with MeOH for 2-3 times to remove the impurities such as excessive glutathione and the remanent species from reducing agent. The NC precipitate was re-dissolved in water to produce a turbid solution, which was subjected to centrifugation to remove majority of insoluble thiolates as a pellet, but minute amounts of free flow thiolate species could be removed by filtering with filter the paper having 0.22 µm pores. Finally, we get the $Au_{18}SG_{14}$ aqueous solution and keep it in the fridge to store for long period of time (≈ 15 days). And we can also get the $Au_{18}SG_{14}$ powder through freeze dried $Au_{18}SG_{14}$ aqueous solution.

Methods: SHIMADZU UV-vis 1900 spectrometer was used for the measurements. UV Spectra were typically measured in the range of 200-1100 nm. A diluted solutions were spotted

on carbon-coated copper grid and was dried in air conditions. Images were collected at 120 keV, which reduces beam-induced damage to the clusters. The photoluminescence spectra and lifetime of the $Au_{18}(SG)_{14}$ and TOA-paired $Au_{18}(SG)_{14}$ clusters were recorded by using Fluo Time 300. The Zetasizer Nano ZSE was used for testing the Au-SG complexes size and distribution.

Phase transfer of highly luminescent $Au_{18}SG_{14}$-TOA Clusters: The two methods were used for the phase transfer of $Au_{18}SG_{14}$ clusters. In the typically phase transfer, two solutions are mixed, namely i) 20 mg TOAB was dissolved in 10 mL toluene, and ii) 25 mL of aqueous $Au_{18}SG_{14}$ solution (half the amount of one synthetic batch) whose pH is adjusted to 9 by the addition of NaOH. The toluene solutions which contain TOAB were added to $Au_{18}SG_{14}$ solutions and stirred it for several minutes. A colorless toluene solution changes to reddish brown indicates the clusters undergone the phase transfer. In another phase transfer method, initially, 25 mg TOAB was dissolved in 20 mL toluene, the 20 mL $Au_{18}SG_{14}$ solutions were added to a beaker, then diluted the solution by 8 times (≈ 160 mL). Next, the toluene solutions which contain TOAB were added to the $Au_{18}SG_{14}$ solutions and vigorously stirred for several minutes results the phase transfer of clusters from aqueous to organic medium.

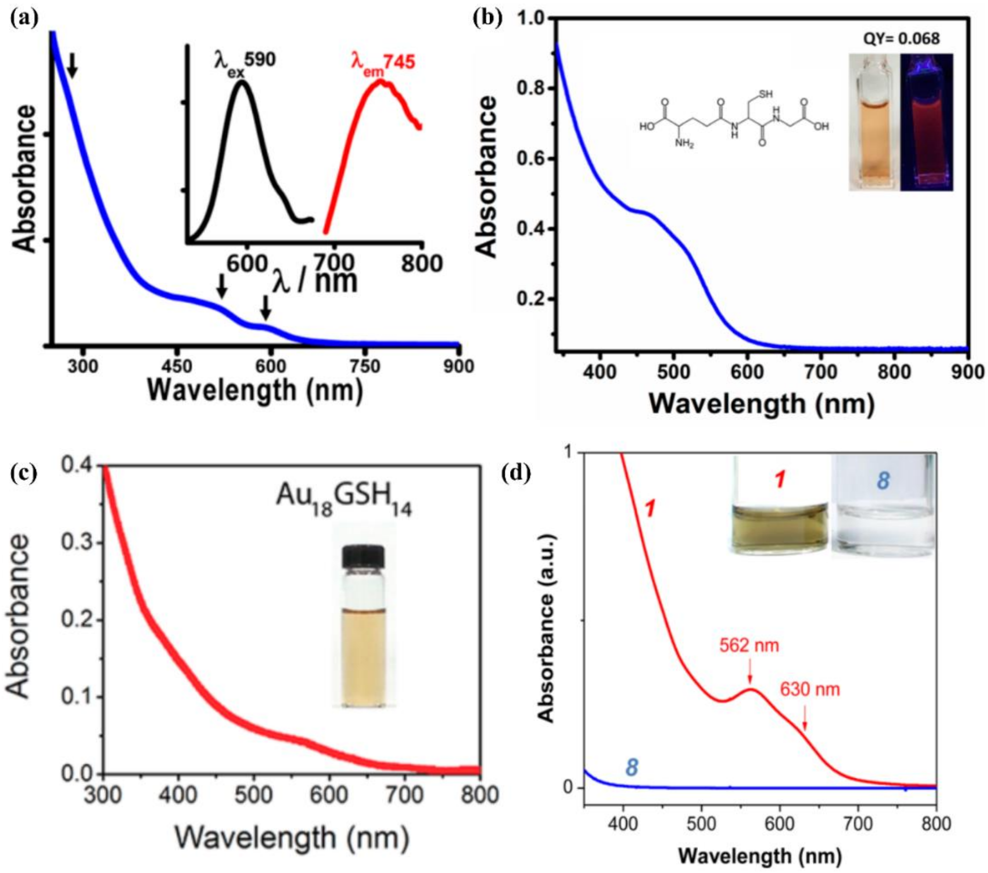

Figure S1. The absorption spectra of the $Au_{18}SG_{14}$ which reported in the literature. (a), (b), (c), and (d) are adapted from the following references (1-4).

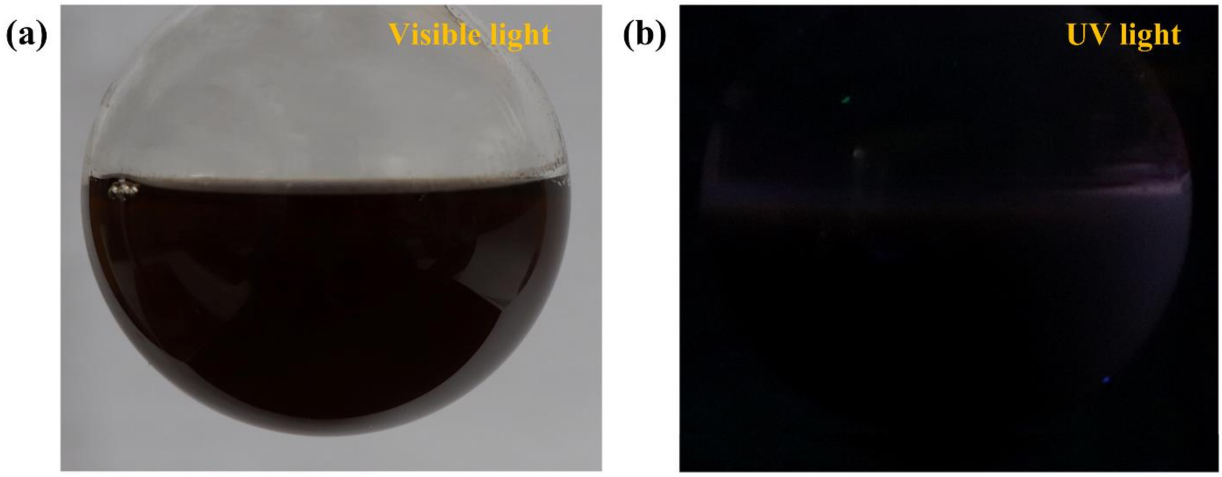

Figure S2. (a), (b) are the digital photograph of the crude reaction solution after the successful completion of the reduction process by $NaBH_3CN$. Herein the synthesis is performed in the absence of methanol *i.e.* the reaction is done in deionized water.

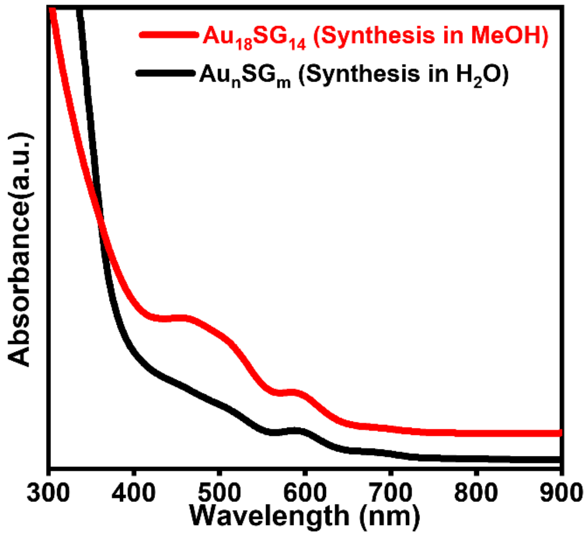

Figure S3. The absorption spectra of the NCs synthesized at two different solvent conditions. i) black trace, pure water, and ii) red trace, excess methanol (water : methanol, 1 : 48).

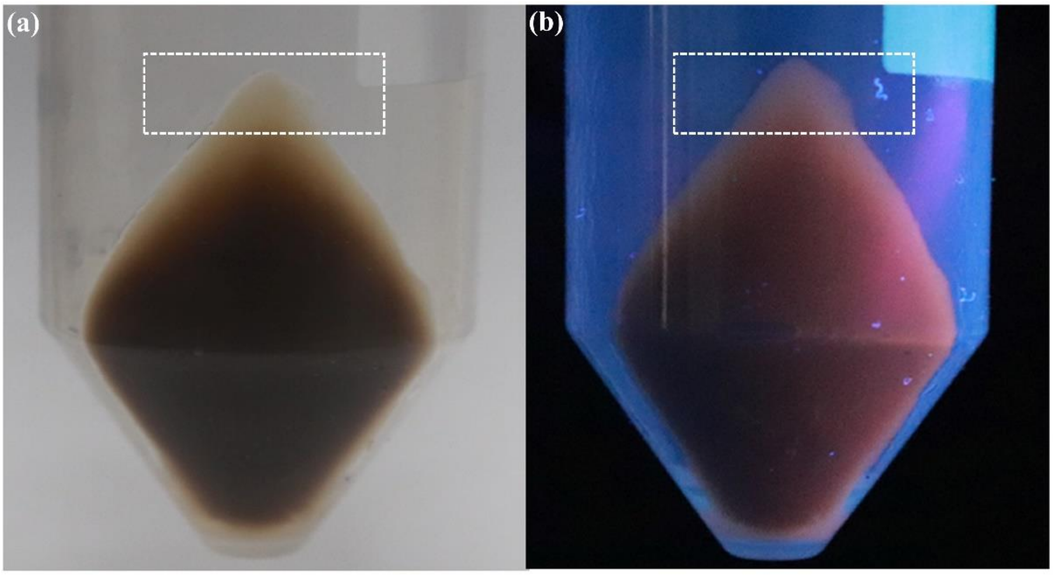

Figure S4. The solid pellet of the crude NCs prepared at 17 °C under visible light (a) and UV light (b). The rectangle dotted lines are marked to represent the white colored solid (in contrast to the middle portion which is brownish color), corresponding to $[Au(I)SG]_x$ sediments solution.

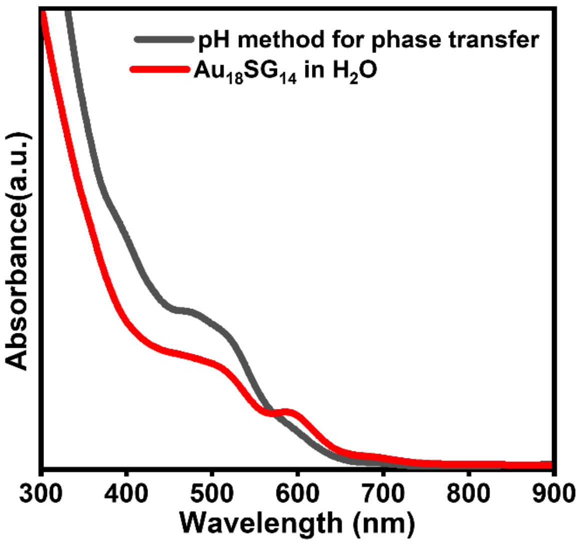

Figure S5. The absorption spectra of the $Au_{18}SG_{14}$ NCs for pH method used for the phase transfer.

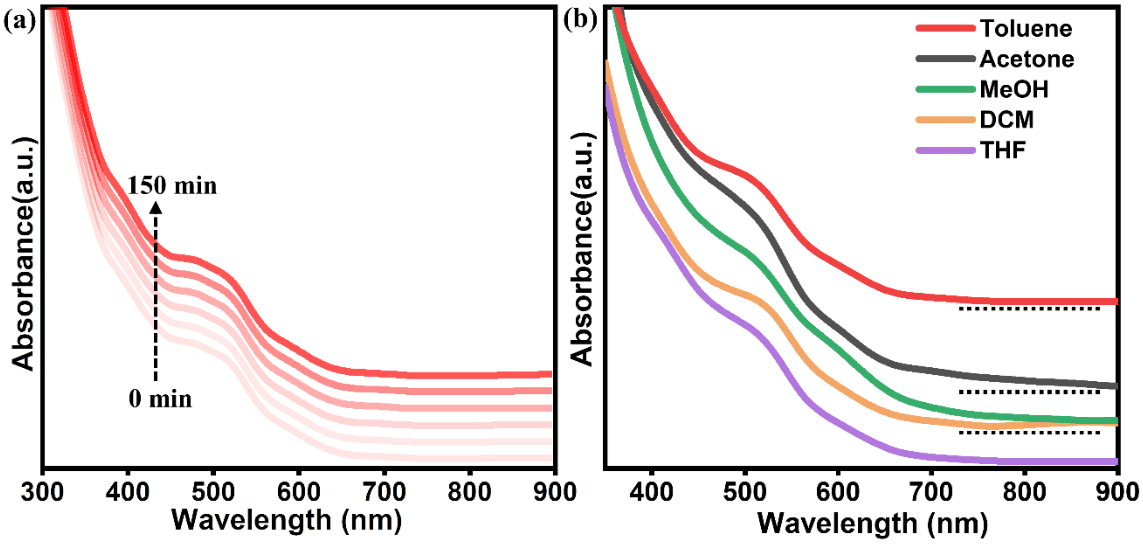

Figure S6. (a) The time-dependent absorption spectra of phase-transferred NCs in toluene were obtained by pH method. (b) The absorption spectra of the phase-transferred NCs clusters in different solvents were obtained by pH method. Note that in pH method, the Au_{18} NCs lost their characteristic absorption peak at 590 nm, which implies the original NCs undergoes structural modifications.

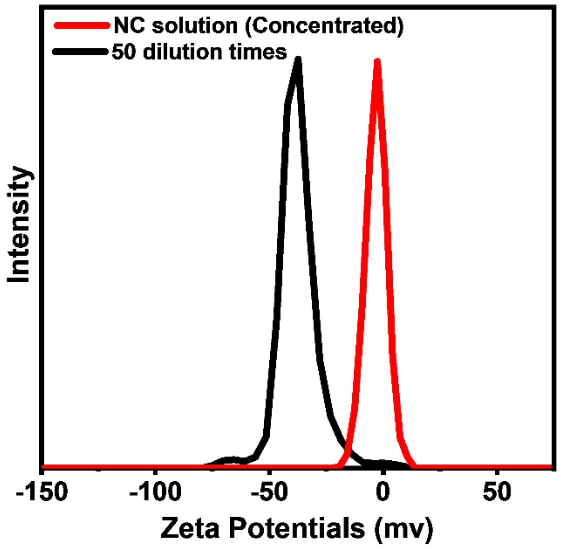

Figure S7. The zeta potential measurement of $Au_{18}SG_{14}$ at two different concentrations.

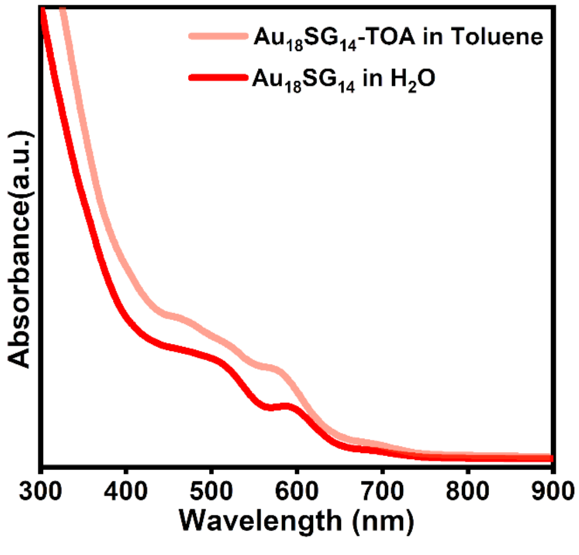

Figure S8. The absorption spectra of the $Au_{18}SG_{14}$ NCs in the dilution method used for the phase transfer.

References

1. Ghosh, A.; Udayabhaskararao, T.; Pradeep, T. *J. Phys. Chem. Lett.* 2012, 3 (15), 1997-2002.
2. Yu, Y.; Yao, Q.; Chen, T.; Lim, G. X.; Xie, J., *J. Phys.Chem.* C 2016, 120 (38), 22096-22102.
3. Stamplecoskie, K. G.; Kamat, P. V., *J. Am. Chem. Soc*. 2014, 136 (31), 11093-11099.
4. Yousefalizadeh, G.; Stamplecoskie, K. G., *J. Phys.Chem.* A 2018, 122 (35), 7014-7022.
